# Hyaluronan and the Fascial Frontier

**DOI:** 10.3390/ijms22136845

**Published:** 2021-06-25

**Authors:** Rebecca L. Pratt

**Affiliations:** Department of Foundational Medical Studies, Oakland University William Beaumont School of Medicine, Rochester, MI 48309, USA; rebeccapratt@oakland.edu; Tel.: +1-248-370-3670

**Keywords:** hyaluronan, hyaluronic acid, fascia, fasciacyte, gliding, connective tissue, HA, extracellular matrix, myofascial pain, densification

## Abstract

The buzz about hyaluronan (HA) is real. Whether found in face cream to increase water volume loss and viscoelasticity or injected into the knee to restore the properties of synovial fluid, the impact of HA can be recognized in many disciplines from dermatology to orthopedics. HA is the most abundant polysaccharide of the extracellular matrix of connective tissues. HA can impact cell behavior in specific ways by binding cellular HA receptors, which can influence signals that facilitate cell survival, proliferation, adhesion, as well as migration. Characteristics of HA, such as its abundance in a variety of tissues and its responsiveness to chemical, mechanical and hormonal modifications, has made HA an attractive molecule for a wide range of applications. Despite being discovered over 80 years ago, its properties within the world of fascia have only recently received attention. Our fascial system penetrates and envelopes all organs, muscles, bones and nerve fibers, providing the body with a functional structure and an environment that enables all bodily systems to operate in an integrated manner. Recognized interactions between cells and their HA-rich extracellular microenvironment support the importance of studying the relationship between HA and the body’s fascial system. From fasciacytes to chronic pain, this review aims to highlight the connections between HA and fascial health.

## 1. Introduction

**Figure 1:**
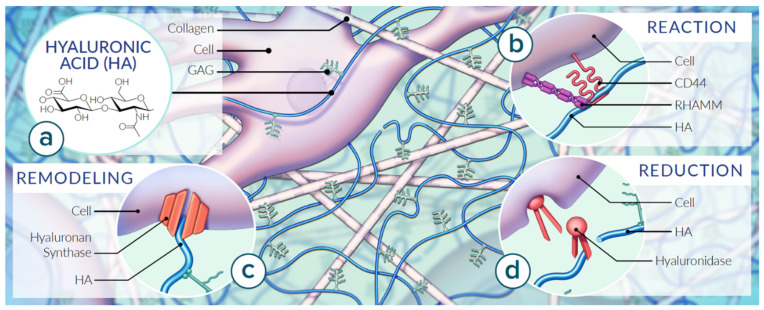
Properties of HA in the context of the ECM of fascia. Matrix organization of HA varies by tissue type reinforced mechanically by types of collagen fibers and managed by tissue-specific cell types. (**a**) HA consists of repeating disaccharide units of D-glucuronic acid and N-acetyl-D-glucosamine structured as a single polysaccharide chain abundantly present in the extracellular matrix (ECM). (**b**) Extracellular HA plays a role in adhesivity via cell receptors. Mechanical properties of HA are dictated by molecular weight, abundance and fluid dynamics. The cluster determinant 44 (CD44) embedded in the cell membrane mediates adhesion, migration and intracellular signaling. The receptor for HA-mediated motility (RHAMM) modifies intracellular signaling. (**c**) HA concentration is coordinated by hyaluronan synthases which is (**d**) balanced by snipping enzymes referred to as hyaluronidases. Based on [17,18,19].

The polysaccharide hyaluronan, built from repeating disaccharide units, first entered the scientific world in 1934 when it was discovered by Meyer and Palmer [1]. Hyaluronan (HYA and HA; synonymous hyaluronic acid and hyaluronate, respectively) is one of a group of polysaccharides typically found in the connective tissues of vertebrates; originally known as acid mucopolysaccharides, they are now labeled as glycosaminoglycans [1,2,3]. Glycosaminoglycans are unbranched single-chain polymers of disaccharide units containing N-acetyl hexosamine and hexose [3]. The second sugar is a hexuronic acid in all except keratan sulphate, which contains galactose instead [2,3]. HA consists of repeating disaccharide units of N-acetyl-D-glucosamine and D-glucuronic acid and is abundantly present in the extracellular matrix (ECM) (Figure 1a). The HA backbone can comprise several thousand sugar molecules [4]. HA is a polyanion that can self-associate as well as bind to water molecules, when it is not bound to other molecules, forming a viscous, Jell-O-like environment [4]. Although HA consists of a single polysaccharide chain similarly to other glycosaminoglycans, it stands out among them because its molecular weight can reach the millions [3]. The sizes of HA polymers range from 5000 to 20,000,000 Da in vivo, and the functions of HA are largely dictated by its size [5]. The high molecular weight HA is found in normal, intact, healthy tissue, where it aids in maintaining normal homeostasis by suppressing cell proliferation, migration, angiogenesis, inflammation, and immunogenicity [6]. In contrast, it has been verified that low molecular weight HA stimulates the motility of tumor cells via binding to CD44 receptors [7,8]. According to studies by Toole et al. (2004), low molecular weight HA oligomers can stimulate endothelial proliferation and migration. It is not completely known whether the tumor cells are responsible for producing these oligomers of HA, but the overall effect may lead to degradation of the normal macromolecular partnership between HA and ECM [9]. Emerging reports have also been published, suggesting that the ability to recognize receptors for the HA-degraded fragments as well as the highly polymerized native HA involves interaction with integrins, Toll-like receptors (TLRs), receptors for HA-mediated motility (RHAMM) and the cluster determinant CD44 (Figure 1b) [10]. CD44 is involved in inflammatory processes, atherosclerosis, and carcinogenesis [11]. RHAMM is responsible for the motility of cells, tissue healing, and carcinogenesis [12,13,14]. The interaction between HA and receptor CD44 affects proliferation, survival, motility, invasiveness, and the chemoresistance of cells. The overexpression of the receptor RHAMM is associated with the promotion of tumor growth and metastasis. Due to the role HA plays in the ECM, modifications to HA have become recognized as an active participant in inflammatory, angiogenic, fibrotic, and cancer-promoting processes [9,15,16].

HA is synthesized by integral membrane proteins through the addition of monosaccharides to the reducing terminal. Unlike other glycosaminoglycans, the primary structure for HA contains no peptide, which is consistent with its synthesis in the plasma membrane rather than the Golgi [3]. HA production is coordinated by hyaluronan synthases and balanced by cleavage enzymes referred to as hyaluronidases (Figure 1c,d). Different sulphated glycosaminoglycans accompany the saccharides for a diverse array of proteoglycan structures [3]. In tissues, HA can occur bound to plasma membranes, aggregated with other macromolecules, or as free polysaccharides [2,3,16].

### HA Structure and Function

In both vertebrates and bacteria, the HA structure is identical [20]. HA can be detected in a large variety of tissues and fluids including, but not limited to, connective, epithelial and neural tissues, where it delivers mechanical stability while acting as a water reservoir and lubricant [2]. An average human body weighing 70 kg contains 15 g of HA [4]. The greatest contributors to overall HA are the dermis (about half of the total HA), as well as the synovial fluid, the vitreous body and the umbilical cord [20]. Recently, debates have been raised regarding HA lubrication behaviors and surface adhesion and the role HA plays as a true boundary lubricant in synovial joints [21]. Despite this ongoing controversy, most studies indicate that a major mechanical role of HA is to maintain the viscosity of synovial fluid. It can do so under physiological conditions with mediated interactions with some or all of the four major components of synovial fluid: other HA molecules, lubricin, lipids and cartilage Type II collagen fibrils [22]. HA is also located in places where lubrication between two surfaces is required, such as the joints, tendon sheaths, the lung pleura, and the heart pericardium. In addition to continuous deformation over time, HA’s ability to rid the body of free radicals also plays an important role in wound healing, ovulation, fertilization, signal transduction, and tumor physiology [20].

The high molecular weight and the semi-flexible polymer of HA are key factors leading to the high viscosity of dilute solutions. HA solutions can display increased viscosity due to mutual macromolecular crowding [23]. Additionally, HA functions include lubrication, water homeostasis, filtering effects, and the location of proteins within the plasma membrane, made possible by the manner in which the network of HA molecules controls and impedes the diffusion of other molecules [3]. The shear rate dependence of the viscosity, and the viscoelasticity of HA solutions, relate to the relaxation time of the molecule, which, in turn, depends on the HA concentration, molecular weight, temperature and pH [24]. High viscosity can additionally affect the lubricating function of HA solutions. In particular, the three-dimensional structure of HA progressively breaks down when the temperature is increased to >40 °C with a resulting decrease in viscosity [24].

When the carboxyl groups of HA are fully ionized at extracellular pH, HA’s osmotic activity is disproportionately high in relation to its molecular weight. This molecular characteristic means that it is capable of intense impact on the movement and distribution of water, and thus, water homeostasis [3]. HA viscosity has a direct relationship with pH [24]. For example, Juel et al. in 1990 and further in 1998 and 2004 demonstrated that pH alterations can change the viscosity of loose connective tissue. Recall that loose connective tissue is an important reservoir of water and salts for surrounding tissues [25,26]. In addition, it also accumulates cellular waste products. Therefore, the biomechanical properties of loose connective tissue can be altered depending upon accumulated lactic acid content after intense exercise. In their studies, it was determined that within a muscle compartment, pH can reach a value of 6.60, resulting an increase of approximately 20% in HA viscosity, with a consequent sensation of momentary stiffness [27].

As early as 1986, a team of researchers reported that HA can influence cell migration by cooperating with the intercellular matrix in cell detachment from the ECM [2]. We have learned that the regulated production of soluble forms of HA receptors, alternately spliced cell surface isoforms, and glycosylation variants of these receptors can also dramatically influence HA binding, ligand specificity, and stimulation of signaling pathways [11,19]. It has been reported that an increase in HA concentration within the surrounding tissue matrix of a neoplasm is associated with increased interstitial pressure, which can constrict the lumen of the supplying blood vessels. This phenomenon often results in impaired vascular function and drug resistance [7,11]. It should be noted that both the HA and its counterpart hyaluronidase have pro- and anti-tumor effects, which are concentration- and origin-dependent (endogenous or oncogenic) [28,29,30]. Further clarification of the molecular mechanisms regulating HA-mediated events will not only contribute greatly to our understanding of a variety of disease processes, but will also offer many new avenues of therapeutic intervention [31].

Tissue HA enters the bloodstream in significant amounts via the lymphatic system of ducts where it is rapidly absorbed by liver endothelial cells and subsequently degraded [2,3]. In humans, the half-life of HA in tissues ranges from 2–5 min to several days [3]. HA levels in blood serum are normally 10–100 micrograms/L, but can be elevated in situations such as cirrhosis, rheumatoid arthritis and scleroderma. This increase in HA could be due either to impaired hepatic uptake or to increased production [2].

There are many excellent reviews on the roles of HA in different fields such as in angiogenesis, reactive oxygen species, HA digestion, cancer, cancer therapeutics, cancer metastasis, chondrocytes, lung injury, wound healing, diabetes, leukocyte trafficking, and immune regulation [11]. This review focuses on specifically highlighting HA in the important field of connective tissue, and how the properties of HA, such as improving tissue hydration and resisting mechanical damage, aid in total body health.

## 2. Cellular Production of HA

It is understood that most vertebrate cells synthesize HA at some point in their natural history [3,32,33]. In the adult form, the synthesis of HA is most strongly expressed in cells of mesodermal lineage, although production can remain active in other cells such as those of the epidermis. In adults, the synthesis of HA is most strongly expressed in cells of mesodermal lineage, although production can remain active in other cells such as those of the epidermis. As stated by Jacobetz et al. (2013), an especially significant increase in the levels of HA can be observed in tumors of epithelial origin. For example, 87% cases of adenocarcinomas of the pancreas have a high expression of HA [7]. Therefore, it is not surprising that HA production increases in proliferating cells [20,33]. The cellular components of fascia are responsible for adapting the extracellular environment in response to a variety of conditions and roles. The ground substance, of which HA is a robust component, is a water-rich gelatinous substance that provides flexibility to tissues, permits the gliding of internal organs, and oversees the transportation of metabolic material [34]. Due to the body-wide distribution of HA-rich ground substance, the variety of HA-producing cells is plentiful; a few of the significant manufacturers have been selected.

### 2.1. Fibroblasts

Fibroblasts reside in close proximity to collagen fibers (Figure 1). Their thin, pale staining processes contain cytoplasm and extend in many directions, blending into the ground substance they synthesize [17]. Fibroblasts have many properties, including the ability to contract and to communicate with one another. Fibroblasts play a key role in the maintenance of the interstitial fluid of the ECM ground substance [35]. When fibroblasts, mesothelial or certain other kinds of cell are plated out in tissue culture, they surround themselves within a few hours with a transparent pool or coating of gel-like material that can be visualized by its impressive ability to exclude cells and other particles, but otherwise cannot be seen with regular histological techniques [3,36]. This material is reduced when treated with hyaluronidase, suggesting that this matrix depends on HA for its integrity; initially, at least, it must represent the newly synthesized polysaccharide translocated to the pericellular space [3,36]. HA is also detectable in normal bodily fluids such as plasma and urine and highly concentrated in fluids with anti-friction properties such as pleural, synovial and peritoneal fluids [16].

### 2.2. Smooth Muscle Cells

Smooth muscle cells have organelles typical of secretory cells. Smooth muscle cells synthesize various types of collagens, as well as proteoglycans and adhesive glycoproteins [17]. HA synthesis capabilities can be repressed or activated in changing circumstances, as in the case of the smooth muscle cell [10]. For example, an increase in TGF promotes smooth airway muscle cells to secrete HA, resulting in the abnormal increased HA levels observed in asthma patients [11]. Strong staining for HA is also evident in the submucosa and ECM of smooth muscle. In a study performed by Hinata (2013), authors suggest that elastic fibers and HA coexist and interact in the submucosa and smooth muscle sphincter sites [37].

### 2.3. Synoviocytes

Synoviocytes are found within the architecture of diarthrodial joints, specifically, the delicate joint lining. The joint lining is divided into two functionally and anatomically distinct layers: the intimal lining layer and the sublining layer [38]. Synoviocytes are mesenchymal cells that populate both layers and display many characteristics commonly associated with neighboring fibroblasts. Their unique surface adhesion molecules and the production of lubricin set them apart from their neighboring fibroblasts [38]. HA, one of the principal components of synovial fluid, is also produced by synoviocytes. Here, HA plays a role in maintaining joint fluid viscoelasticity, lubrication, and acts as a shock absorber. HA also supports cartilage in being resistant to compression [39,40].

### 2.4. Fasciacytes

**Figure 2:**
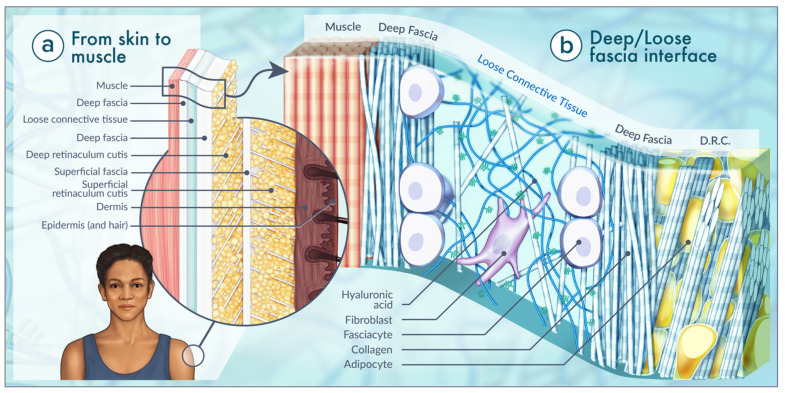
Characteristics of the human fascial system, considered in its three-dimensional continuity. Fascia is composed of soft, collagen-containing loose and dense fibrous connective tissue that permeates the body. (**a**) From the skin to the deepest plane, we find the epidermis, dermis, superficial fascia, divided into two fibroadipose layers (superficial retinaculum cutis (SRC) and deep retinaculum cutis (DRC)), deep fascia, a loose connective tissue layer, deep fascia and skeletal muscle. (**b**) In 2020, Purslow established that the configuration of epimysium closely mirrors the organization of aponeurotic fascia with loose connective tissue intervening between two or possibly three sublayers of deep fascia. The main constituents of the loose connective tissue are water, ions, and glycosaminoglycans, with a robust prevalence of HA. Fasciacytes are at home in the loose connective tissue interface between layers of denser, deep fascia covering skeletal muscle of the extremities and regions of the trunk. Based on [17,41,42,43,44].

One of the biggest breakthroughs to date in the discipline of HA research was reported in 2018 by C. Stecco’s team [41]. A new class of cells termed fasciacytes, not previously recognized in extracellular tissue, was observed as a single layer that stained prominently with Alcian blue. Fasciacytes are not fibroblasts; they are described as fibroblast-like cells located in the loose connective tissue interface between layers of neighboring tissues. As with fibroblasts, fasciacytes are positive for vimentin and negative for the CD68 marker, indicating that they originate from the monocyte–macrophage lineage. Unlike fibroblasts, these cells have a distinct morphology which is much plumper with central nuclei. Fasciacytes’ unique location along the planes of fascial layers also supports the distinctions in cell type (Figure 2b). Using RT-PCR, C. Stecco et al. reported the expression of hyaluronan synthase 2 (HAS2) mRNA by these fasciacytes. Based on this summary of findings, the authors postulate that these cells are dedicated to producing the HA-rich ECM found in the continuum of connective tissues [41,42].

## 3. HA in the Extracellular Matrix (ECM)

Our knowledge of the ECM composition and function has increased considerably over the last decade. Numerous studies have demonstrated that the ECM is a source of signals affecting cell adhesion, shape, migration, proliferation, survival, and differentiation [10]. HA is the most abundant polysaccharide of the ECM of connective tissues [16,23]. HA is traditionally regarded as biological Jell-O, or ground substance, which participates in lubricating joints and holding together connective tissues (Figure 1) [9,45]. Specific derivatives of HA and the water it attracts, referred to as the interstitial fluid, comprises the majority of the ECM ground substance [46]. The distribution of cell types within the ECM modifies HA and ultimately the function of the ECM, and therefore fascia. By manipulating the ground substance (glycosaminoglycans, HA, proteoglycans) as well as the fiber density and direction, the connective tissue type along the fascia continuum can vary [45].

When examining the components of the ECM, a network of fibers (mostly collagen and or elastic microfibrils) can be observed; these are responsible for the three-dimensional scaffold that functions as the structural framework of organs [46]. Due to their complex patterning, fibers of the ECMs exhibit multifaceted mechanical behaviors including viscoelasticity (defined as a time-dependent response to loading or deformation), as well as mechanical plasticity and nonlinear elasticity [46,47]. An impressive characteristic of HA is its ability to interact with many binding proteins such as aggrecans which are found abundantly in the body’s ECM. For example, the family of proteins called the hyaladherins specifically recognize the HA structure. Interactions with hyaladherins bind HA with proteoglycans to stabilize the framework of the ECM [3]. Recent work by Chaudhuri (2020) has revealed that the viscoelasticity of the ECM can regulate fundamental cell processes [47]. Their results provide remarkable insights into cell–ground substance interactions and how these interactions can differentially regulate various mechano-sensitive molecular pathways in cells.

Considerable study over the past two decades has established that ECM elasticity or, in contrast, stiffness, affects fundamental cellular activities, including cellular spreading, growth, proliferation, migration, and differentiation [47]. Modifications in the makeup of the ground substance of the ECM have been shown to promote cell motility, adhesion, and proliferation [48]. These processes require massive cell movement and tissue reorganization that are often accompanied by elevated levels of HA. Therefore, HA and its binding play an important role in morphogenesis, wound repair, inflammation, and metastasis. Many of the effects of HA are mediated through cell surface receptors, three of which have been molecularly characterized, namely, CD44, RHAMM, and ICAM-1 [19]. The interactions between cells and their extracellular ligands within an HA-rich ground substance requires further attention.

A variety of extracellular signals regulate the expression of HA and its receptors, concluding that HA-receptor signaling is a tightly controlled protocol. “Binding of the HA ligand to its receptors triggers signal transduction events which, in concert with other ECM and cytoskeletal components, can direct cell trafficking during physiological and pathological events.” [19]. For example, interaction between a HA receptor and extracellular polysaccharide has been connected with cell locomotion and cell migration across the ECM [33]. These HA-mediated signals are propagated by the activation of protein phosphorylation cascades as well as the release of cytokines, resulting in the stimulation of cell cycle proteins [19]. These precise interactions between cells and their microenvironment further support the importance of examining the relationship between HA and the body’s fascial system.

## 4. A Home for HA: Fascia

Although fascia may not be the most glamourous organ system, it is one of the most influential. The fascial system is now being recognized with roles in pathology, fluid movement and proprioception [45]. The fascial system interpenetrates and surrounds all organs, muscles, bones and nerve fibers, endowing the body with a functional structure, and providing an environment that enables all bodily systems to operate in an integrated manner [49]. Studies on aqueous humors, middle ear secretion, amniotic fluid, lung lavage fluid, urine, skin diseases, and cancer have led the way for research in the fascia frontier [2]. The interest in the role of HA in fascia stems from the fact that it is widely available, fully resorbable, and biocompatible [20].

### 4.1. Definition of Fascia

By general definition, fascia is the ECM plus cells, organized as a three-dimensional network that surrounds, supports, suspends, protects, connects, and divides muscular, skeletal and visceral components of the body [49,50]. It is the soft tissue component of the connective tissue system that permeates the human body, forming a whole-body continuous three-dimensional matrix of structural support referred to as fascia [34]. Fascia and its collagen and cellular diversity form a continuum throughout the body. It is this continuum itself that assures the health of the body. The fascial system has no discontinuity in its path, with layers of different characteristics and properties overlapping [49,50]. Fascia interacts with various other structures: muscles, nerves, vessels, and organs [51,52]. It is the continuity of fascia between regions that enables us to appreciate its key role in coordinating not only muscular activity but the body-wide maintenance of water [46].

The diversity of existing categories of fascia reflects not only the complex architecture of fascia itself, but also the diverse group of professionals working in different fields. Schleip et al. (2012) describe three separate fascia terminologies, which if appropriately applied, allow one to discuss discipline distinctions as well as tissue continuities [51].

### 4.2. Anatomy of Fascia

There are two emerging definitions of fascia: one that anatomically defines fascia as “a sheath, a sheet or any other dissectible aggregations of connective tissue that forms beneath the skin to attach, enclose and separate muscles and internal organs” and the “fascial system” that highlights the more functional properties of the three-dimensional fascia which infiltrates all body systems so that they operate in an integrated manner [34]. The anatomical definition of fascia is used in this review (Figure 2a). This definition is based on the established histological observation of connective tissue as one of the four basic tissue types. It is widely acknowledged that connective tissue is taxonomically sub-divided into three broad categories: embryonic connective tissue (mesenchyme, mucous connective tissue), connective tissue proper (loose and dense connective tissue), and specialized connective tissue (blood, bone, cartilage, adipose tissue, hemopoietic tissue, lymphatic system) [17,49,53]. Based on this taxonomy, fascia is generally placed in the category of connective tissue proper, although its specific sub-identification as loose connective tissue, or ‘regularly’ or ‘irregularly’ arranged dense connective tissue is often left undefined because the fascial system is observed as a continuum of connective tissue proper. Therefore, using a histological definition, the fascial network comprises all, rather than just one, types of connective tissue proper (e.g., areolar/loose, dense regular/irregular and adipose) [17,49,53,54].

The fascia incorporates a spectrum of anatomical objectives such as adipose tissue, visceral adventitia and neurovascular sheaths, muscle aponeuroses, deep and superficial fascia, joint capsules, ligaments, tendons, membranes, meninges, myofascial expansions, periosteum, retinacula, septa, tendons, and all the intramuscular and intermuscular connective tissues including endo-/peri-/epimysium [17,49,53,54]. The nomenclature of deep fascia is given to structures such as the fascia lata, thoracolumbar fascia, plantar and palmar fascia, along with regional specializations of deep fascia such as retinacula and fibrous pulleys [46]. The nomenclature of superficial fascia is given to structures such as lamina propria, Camper’s fascia, loose connective tissue interface between perimysium, fundiform ligament, subcutaneous tissue, hypodermis and buccal fascia. Both the superficial and deep fascia listed above all have functional relationships with nerves, vessels and muscles [42,55].

The deep fascia that surrounds skeletal muscle cells (endomysium) and skeletal muscle fascicles (perimysium) and named muscle bellies (epimysium) is essential for translating muscle movement at a joint [17]. This muscular deep fascia contains high levels of HA in the interface between sublayers of the endomysium, perimysium and epimysium [24,43]. Perivascular and perineural connective tissues were also distinctly HA-positive [56]. HA is ubiquitous in the fascia, particularly in that of loose connective tissue surrounding muscle bundled by deep fascia (Figure 2b). Recall that the epimysium covers named skeletal muscle and connects the compartments of skeletal muscle to their bony attachments. Epimysium is continuous with the connective tissue adhering to the bone, referred to as the periosteum [34]. Purslow published that the configuration of epimysium closely mirrors the organization of aponeurotic fascia with loose connective tissue intervening between two or possibly three sublayers of deep fascia [44]. In 2001, McCombe et al. confirmed that HA is located in considerable amounts at the interface between layers of the deep fascia and the epimysium covering the surface of named skeletal muscle. Here, HA acts as a lubricant for deep fascia to glide over muscle epimysium [43]. Deep fascia consists of 2–3 layers of parallel collagen fiber bundles, with each layer having a mean thickness of 277 μm (±SD86.1 μm). The collagen fibers of adjacent layers are oriented in different directions forming angles of 75–80° [24]. Each layer comprising the deep fascia layer is further separated from the other by a thin layer of loose connective tissue (mean thickness 43 ± 12 μm) that enables the sliding of the several layers on neighboring ones. This organization enables a fibrous layer to function independently. This anatomy is only able to perform properly if all the connective tissue elements, such as ground-substance, are appropriately distributed to allow gliding [24].

## 5. HA and Fascial Gliding

Deep fascia is more than a tough barrier structure of collagen and elastin. It is a metabolically active tissue layer which provides protective functions as well as the recently revealed contributions to gliding [57]. From a histological perspective, deep fascia supports an abundance of cells and nervous tissue working to adapt fascia for various metabolic and mechanical conditions [34]. Deep fascia has parallel longitudinal collagen bundles and rudimentary elastic laminae, giving it both high tensile strength and elasticity. HA occurs between deep fascia and muscle, facilitating gliding between these two structures, and also within the loose connective tissue of the fascia, guaranteeing the smooth sliding of adjacent fibrous fascial layers [41]. At the interface between the deep fascia and the muscle, where fasciacytes are present, the tissue establishes a lubricating layer of HA [41].

The deep fascial layer of densely packed collagen bundles and elastin fibers has HA concentrated on its inner surface, which is in contact with the underlying muscle epimysium. Solutions of HA can be highly viscous, thus affecting the movement of HA-containing fluid layers within and underlying the deep fascia [23]. However, if the dense connective tissue of the epimysium surrounding the muscle is disrupted, the fascia does not retain a distinct interface and does not maintain a gliding layer over the muscle [43].

A study from Fede (2018) quantified, for the first time, the HA content of human fascial samples obtained from a variety of anatomic sites. The authors demonstrated that the average amount of HA varies across anatomic sites, specifically sites that allow various degrees of fascial plane sliding and gliding [58]. For example, the fascia lata of the anterior thigh has HA 35 μg/g of fascial tissue, which is similar to that of the anterior abdominal rectus sheath (29 μg HA/g of fascial tissue). However, in regions of the body where lubrication is not required, such as types of fascia adherent to muscle, they contain far less HA per gram of fascial tissue (6 μg). Examples of these anatomic sites are in the fascia overlying the trapezius and deltoid muscles of the shoulder. The most abundant HA was not surprisingly located in synovial joints. In the fascia associated with mobile joints, such as in the retinacula of the ankle where greater degrees of sliding between fascial layers must occur, the HA increases to 90 μg/g of fascial tissue [58].

A study looking at the lubrication of fascia and HA was reported by Roman (2013). In this study, the authors utilized the squeeze film lubrication theory of fluid mechanics for flow between two plates, as well as the Navier–Stokes equations to quantify the frictionless movement of connective tissue and HA [59]. They aimed to explore the relationship between constant sliding, perpendicular vibration, and tangential oscillation, and the flow of HA immediately below the fascial layer. What they determined is that the fluid pressure of HA increased substantially as fascia was deformed during manual therapies. This variation of pressure caused HA to flow near the edges of the fascial area under manipulation, and this flow resulted in greater lubrication [59].

The influence of HA in fascial gliding can also be observed with the histochemical distribution of HA in microscopically thin layers of loose connective tissue between individual layers of deep fascia. In the study by the C. Stecco (2011), samples of deep fascia along with the underlying muscles were taken from the neck, abdomen and thigh of unembalmed cadavers. Samples were stained with hematoxylin–eosin, Azan–Mallory, Alcian blue and a biotinylated HA-binding protein specific for HA. The stained deep fascia revealed a layer of HA between fascia and the muscle within the loose connective tissue dividing the dense irregular layers [55]. It was postulated that the HA within the deep fascia facilitates the gliding of two adjacent fibrous fascial layers, thus encouraging normal function between layers of deep fascia. In contrast, if the HA adopts a more packed configuration, or if the loose connective tissue alters its HA density, the behavior of the entire layer of deep fascia and the underlying muscle become negatively affected [55].

In summary, HA found between neighboring fascial layers promotes fascial gliding which is facilitated by the presence and activity of fasciacytes. Changes in the concentration, molecular weight, or even covalent modification of HA, as well as changes in binding interactions with other macromolecules, can have dramatic effects on the sliding movement of fascia [23].

## 6. HA and Myofascial Pain

Understanding the connective tissue matrix of fascia, specifically HA, together with the mechanical forces allowed and limited by fascial planes can help uncover specific treatment modalities to relieve chronic pain syndromes [24]. Complete, long chains of HA function differentially compared to short HA chains. Short HA chains have the ability to self-stick. This self-association generates a variety of intermolecular aggregate arrangements and can be observed between surfaces such as fascial sheaths and muscle fascicles [60]. By altering HA concentration and/or length, HA chains can entangle into complex nets, leading to alternate hydrodynamic properties and unconventional visco-elastic properties that may result in myofascial pain [6].

HA is essential for two fascial structures gliding across each other; therefore, the regulation of HA could affect the functions of fascia implicated in myofascial pain [41]. Myofascial pain syndrome (MPS) is described as the muscle, sensory, motor, and autonomic nervous system symptoms caused by the stimulation of myofascial trigger points. On the basis of published literature, modifications in collagen fiber composition, in fibroblasts, or in ECM composition have been postulated to result in MPS. In a manuscript published by A. Stecco et al. (2013), the authors summarize developments within the biology of MPS, specifically, matrix-rich HA and the implications of HA alterations leading to viscosity, adhesions, or an increase in loose connective tissue [6].

### 6.1. Immobility

Movement stimulates HA production and turnover, whereas immobility can increase the concentration of HA without effective HA recycling, increase the viscosity, and reduce the lubrication and gliding of the layers of connective tissue and muscle [23]. HA-mediated inflammation can further increase the viscosity of HA-containing fluids if the HA is modified via covalent attachment of the heavy chains derived from inter-α-inhibitor. Hyaluronidase hydrolyzes HA, thus reducing its molecular weight, and in some cases lowering the viscosity of the ECM fluid [23]. The viscoelasticity of HA solutions correlates with the relaxation time of the HA molecule, which, in turn, depends on the HA concentration and molecular weight. It is hypothesized that immobility may reduce fascial gliding and, in turn, a person’s range of motion. Over time, these alterations in HA can modify both muscle structure and function. Due to the viscoelastic properties of HA, it has a high affinity for the opposing cartilage surfaces of joints. In 1963, Dintenfass reported that the viscosity of HA is reduced by loading conditions and that rest and “unloading” facilitates a return to a more viscous state of ground substance [61].

An increase in the production of HA is the body’s initial effort to increase the gliding efficiency between two surfaces, much like that of a synovial joint [24]. If HA is within thin connective tissue layers the HA chains can entangle, contributing to the hydrodynamic properties of the solution [60]. In addition, Tadmor et al. (2001) showed that when HA is organized into layers, viscosity increases considerably as the distance between the two surfaces increases. This increased viscosity within the loose connective tissue may potentially cause inhibited gliding between the layers of deep fascia collagen fibers [62]. This increase in overall fascial thickness may be perceived by patients as an increase in fascial stiffness and pain.

Placing this information into the bigger picture of fascial relationships reinforces the concept that modifying HA at the interface between loose connective tissue and layers of deep fascia may play an essential role in the etiology of pain associated with movement [24,55].

### 6.2. Inflammation

Once thought to be just a structural molecule in the ECM, HA is now being considered a chief regulator of a variety of inflammatory responses [63]. Increasingly over the years, HA has become recognized as an active participant in inflammatory, angiogenic, and fibrotic processes. Inflammation is correlated with an accumulation and turnover of HA polymers by multiple cell types. It is also known that HA and its binding proteins regulate the expression of inflammatory genes along with the recruitment of inflammatory cells and the release of inflammatory cytokines. A growing body of evidence suggests that cell responses are HA molecular weight-dependent. This is because HA fragments, generated by multiple mechanisms, provoke cellular responses distinct from normal high molecular weight HA [15,63,64]. In pathological states such as inflammation, surges in the amount of short HA chains in the ECM can act as a poor prognostic factor in many cancers [9,15,16].

High molecular weight HA displays anti-inflammatory and immunosuppressive properties, whereas low molecular weight HA is a potent proinflammatory molecule [64]. Recall that the sizes of HA polymers range from a low molecular weight of 5000 to 20,000,000 Da for high molecular weight in vivo [5]. Observations by Trigging et al. (2011) revealed the role of molecular weight (low and high) HA in the activation of innate immunity. The authors reported that the molecular weight of HA influences the degree of inflammatory response and damage vs. cell protection and repair via the induction of Th1 responses that are mediated by Toll-like receptor interactions. Their findings emphasize the role of HA in the treatment of rhinitis and chronic rhinosinusitis [65]. In another example, Chrostek (2018) has shown that HA concentration varies in rheumatic diseases. In rheumatoid arthritis patients, alterations in HA concentration reflect the activity of inflammation and, in turn, pain associated with rheumatic diseases [66]. The activation of small HA fragments has also been implicated in cartilage erosion where fragments produce a large number of damaging intermediates resulting in cartilage destruction [10].

**Figure 3:**
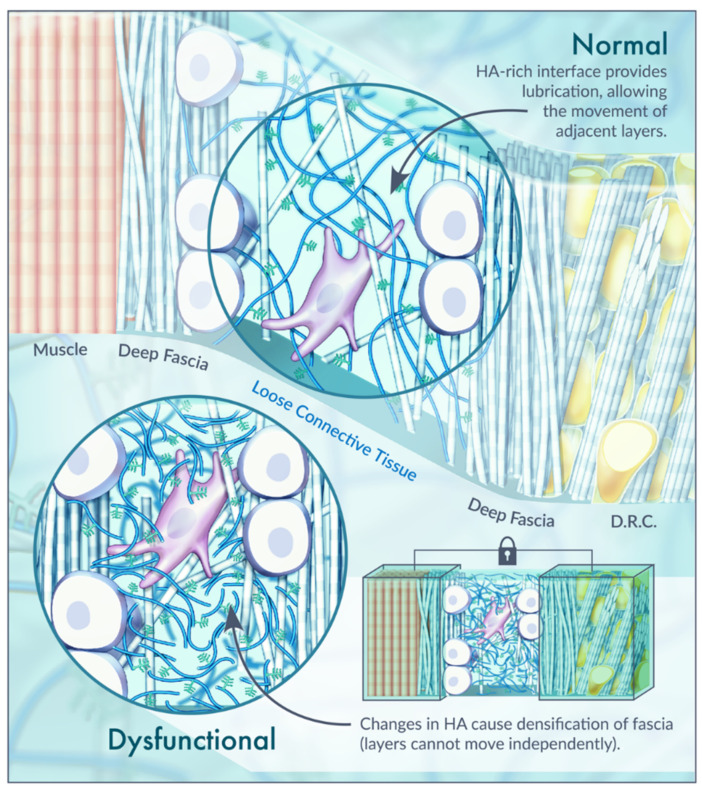
Normal vs. dysfunctional fascial interface. Illustration of the HA-rich interface between layers of dense connective tissue in normal and dysfunctional scenarios. Each of deep fascia layer is separated from the other by a thin layer of loose connective tissue with normal HA (mean thickness 43 ± 12 μm) that permits the sliding of the several layers upon neighboring ones. When HA is found as short HA chains, its small fragments become adhesive rather than lubricating and the distribution of lines of force within the fascia becomes distorted. This is referred to as the densification of fascia, represented by the lock. The tissue layers around the densification site can be a focal point of intense mechanical stress. Based on [6,24,67,69].

In conclusion, an important component of pain therapy is to reverse these changes in HA. When the HA becomes adhesive rather than lubricating, the distribution of lines of force within the fascia become distorted. This is referred to as the densification of fascia [67]. Thickening and densification of the loose connective tissue and its ECM correspond to the reduction in or loss of fascial sliding ability [68]. If loose connective tissue is lost or its density is altered, the behavior of the fascia and underlying muscle becomes compromised (Figure 3) [41]. One key ingredient affecting fascia density appears to be the status of HA [24]. Diet, exercise, or overuse syndromes that result in negative modifications to the loose connective tissue can lead to this distortion of the loose connective tissue between the layers of deep fascia, causing fascial densification. Due to the properties of HA within the ECM, it is theorized that this alteration is reversible through modifications of temperature, pH and mechanical strain (such as massage) [24,67]. A. Stecco et al. (2013) also suggest modifications to viscosity, including a reversal of the aggregation of the HA fragments using increased temperature and local alkalization. The authors hypothesized that these alterations can be accomplished with massage, manipulation, or physical therapies causing disaggregation of the pathologic chain–chain interactions through increases in the subcutis temperature [6]. Validity is therefore provided for massage and other forms of body work that often provide relief for myofascial pain.

## 7. Clinical Application of HA

### 7.1. Manual Therapy

Studies suggest that fascia reorganizes itself along the lines of tension imposed or expressed in the body. This may potentially create restriction between all structures enveloped by fascia itself with mechanical and physiological effects as a consequence of reduced gliding facilitated by HA [50].

From an osteopathic perspective, fascial techniques aiming to release such tensions, decrease pain and restore function are based on various studies that have explored the viscoelastic properties of HA in the connective tissue [50]. More research is needed to understand the flow characteristics of HA during motions used in osteopathic manipulative treatment and other manual therapies [59].

Due to different anatomical locations and to the qualities of the deep fascial tissue, it is important to recognize that different modalities of approach have to be taken into consideration when considering treatment options [52]. Based on the fact that the superficial fascia is closer to the surface than the other layers of fascia, effective treatment can be achieved with light massage or with treatment modalities that use large surfaces to distribute temporary friction in the first layers of the subcutis [52]. For example, gentle pressure applied gradually will allow fascia to rearrange focal adhesions and macromolecule complexes to return to their healthy positions [54,70]. C. Stecco et al. (2016) demonstrated that symptoms related to dysfunctions of the lymphatic system, superficial venous drainage system, as well as thermoregulation are closely related to dysfunction of the superficial fascia in which they are found [52]. Examples of this dysfunction can include, but are not limited to, alterations to a person’s mechanical coordination, proprioception and balance. Myofascial pain or cramps seem to relate more to deep fascia (epimysium) dysfunction. The dense connective tissue organization of deep fascia therefore requires treatment that generates enough pressure to reach the surface of muscles. For this reason, the use of small surface tools and manual deep friction with the knuckles or elbows are indicated [52]. The osteopathic treatment of the fascia involves several techniques such as these, each aimed at allowing the various layers of the connective system to slide over each other, improving dysfunction [35]. Several manual and physical approaches have been proposed to improve myofascial function after traumatic injuries [6].

### 7.2. HA and Treatment

The biocompatibility and negligible negative side effects of HA makes it one of the more readily available compounds used throughout many fields of medicine in the 21st century [20]. Despite the vast number of published studies using HA in medical treatments, it continues to be challenging to distinguish different types of HA and their properties in order to enhance physicians’ clinical practice in terms of the fascial application of different types of HA for various treatments [71]. The following are offered as a small sample size of fascia-focused treatments using various molecular weights and HA concentrations.

The most significant cause of intervertebral disc (IVD) degeneration is the deprivation of HA-linked aggrecans and subsequent water loss present in the ECM of the annulus fibrosus. A recently published manuscript by Bhattacharya (2020) reports that compressive loading causes intensive coiling of HA, which traps more water and aids in bearing compressive loads. The authors indicate that the hydration level of the IVD is strongly influenced by the HA molecules and water molecules in the ECM, which in turn impact the nanoscale mechanics of the annulus fibrosus. An avenue for exploration exists between HA treatment and IVD degeneration [57].

Plantar fasciopathy is the most common cause of plantar heel pain and is considered to be a type of enthesopathy. In 2018, Kumai et al. published a report stating that high molecular weight HA was an effective treatment with no serious adverse drug reactions for plantar fasciopathy. This treatment contributed to the alleviation of pain in patients with plantar fasciopathy and improvement in their activities of daily living [72].

A research study by Chin et al. in 2011 demonstrated that HA augmentation can alter a host’s response to an ECM [73]. In this study, the authors investigated modified high molecular weight tyramine-substituted hyaluronan (TS-HA) to regulate inflammation and fibroblast recruitment into surrounding ECM. Chin et al. (2012) further reported usage for biomaterial scaffolds derived from the ECM to treat torn tendons of the rotator cuff muscles with TS-HA. To observe changes in fascia scaffolding, these experiments were carried out in the rat abdomen. One of their many interesting findings was that TS-HA-treated fascia with crosslinking exhibited reduced stiffness and a higher transition strain than water-treated controls, not only after implantation, but also at time zero. The particular TS-HA treatment employed in this study decreased the low-load elastic mechanical properties of fascia ECM [74]. Augmentation scaffolds derived from human fascia lata ECM may be especially appropriate for tendon repair because of the chemical, structural, and material properties of fascia. As a result, the mechanical properties of treated fascia could be maintained to an extent that prevents or limits the re-tear of rotator cuff muscle tendons over the course of soft tissue healing [74].

Finally, in vocal fold scaring when the lamina propria fascial layer is insulted or deficient, this negatively impacts the viscoelastic properties of the ECM. Use of a polymer such as HA appears most promising for lamina propria replacement therapy because it has the optimal viscoelasticity and also plays a role in the maturation and maintenance of vocal fold fascia [75].

## 8. Conclusions and Perspective

HA is an essential molecule in the ECM and therefore the body’s fascia. The numerous functions of HA depend on its size, location, and interactions with its binding proteins. Although significant progress has been made in translating the biochemical properties of HA in human tissue, researchers must continue to investigate strategies of targeting the HA network for human myofascial dysfunction. This knowledge requires current research on inflammation, fibrosis, and cancers using both high molecular weight HA and chemically modified HA preparations, HA inhibitors, HA blocking peptides, and hyaluronidases to be extrapolated to the discipline of fascia. It is the author’s hope that this brief review will serve as a foundation for communication and conversation across the various research fields exploring the highly dynamic and critical roles of HA and body health.

## Data Availability

Not applicable.

## Abbreviations

HA	hyaluronan
CT	connective tissue
ECM	extracellular matrix
CD44	cluster determinant 44
RHAMM	receptor for HA-mediated motility
MPS	myofascial pain syndrome
IVD	intervertebral disc
Th1	T helper 1 cell
TS-HA	tyramine-substituted hyaluronan
DRC	deep retinaculum cutis
SRC	superficial retinaculum cutis
